# Supplementary material for: STRetch: detecting and discovering pathogenic short tandem repeat expansions
Source: Genome Biol. 2018 Aug 21;19:121. doi: 10.1186/s13059-018-1505-2 (PMC6102892; doi:10.1186/s13059-018-1505-2)
Supplement: Supplementary file 1 — Supplementary tables, figures and methods STRetch_additional_file_1. (PDF 1095 kb) [file 13059_2018_1505_MOESM1_ESM.pdf]

Supplementary Materials

Table S1: Comparison of allele sizes (number of repeat units) called by STRetch, LobSTR, HipSTR and ExpansionHunter in the 10 individuals with known STR alleles.

Sample	disease	gene	repeat unit	type	position	allele ref	allele PCR	allele STRetch	allele LobSTR	allele HipSTR	allele Expansion Hunter
Sample 1	SCA1	ATXN1	CAG	coding	chr6:16327865-16327955	30.3	51	50.5	31.3/31.3	36.3/39.3	31/70
Sample 2	Unaffected relative of sample 1	ATXN1	CAG	coding	chr6:16327865-16327955	30.3	29/32	32.2	30.3/35.3	30.3/35.3	30/35
Sample 3	SCA3	ATXN3	CAG	coding	chr14:92537355-92537396	14	73	65.4	28/28	no call	25/70
Sample 4	SCA6	CACNA1A	CAG	coding	chr19:13318673-13318712	13.3	22	no call- 0 STR reads in all samples	11.3/24.3	11.3/22.3	11/22
Sample 5	SBMA	AR	CAG	coding	chrX:66765159-66765261	33.3	41	43.3	no call	no call	52/55
Sample 6	SBMA	AR	CAG	coding	chrX:66765159-66765261	33.3	47	35.1 (0 STR reads)	no call	no call	57/63
Sample 7	FTDALS1	C9orf72	GGGGCC	intronic	chr9:27573482-27573544	10.8	>50	41.5	no call	no call	12/980
Sample 8	DM2	ZNF9	CCTG	intronic	chr3:128891419-128891502	20.8	>75	38.4	14.8/14.8	no call	14/48
Sample 9	DM1	DMPK	CAG	non-coding	chr19:46273462-46273524	20.7	>150	79.3	5.7/5.7	20.7/5.7	5/140
Sample 10	FRDA	FXN	GAA	intronic	chr9:71652203-71652205	6	~850	17.7*	No call	No call	83/117

Table S2: Raw data for sensitivity, specificity and FDR calculations. The ten true positive samples (Samples 1-10) and eight of the 97 control samples (Controls 1-8) were tested using STRetch at 22 known pathogenic loci (FRDA was omitted as it is not annotated in the reference genome). Cells contain the STRetch p-values (using reference controls) for all significant hits (p<0.05).

	Sample 1	Sample 2	Sample 3	Sample 4	Sample 5	Sample 6	Sample 7	Sample 8	Sample 9	Sample 10	Control 1	Control 2	Control 3	Control 4	Control 5	Control 6	Control 7	Control 8
CCHS_PHOX2B																		
DM1_DMPK									1.13E-32									
DM2_ZNF9								4.05E-16										
DRPLA_ATN1																		
FRA12A_DIP2B																		
FRAXE_AFF2																		
FTDALS1_C9orf72	5.08E-08	3.88E-07				5.30E-08	1.20E-06	9.91E-03					3.57E-04	1.07E-05				
FXTAS_FMR1																		
HD_HTT									1.12E-04									
HDL2_JPH3																		
OPMD_PAPBN1																		
SBMA_AR					5.19E-05													
SCA1_ATXN1	2.46E-14																	
SCA2_ATXN2																		
SCA3/MJD_ATXN3			4.15E-09		5.75E-09				3.20E-08									
SCA6_CACNA1A																		
SCA7_ATXN7																		
SCA8_ATXN8/ATXN8OS											4.24E-24							
SCA10_ATXN10																		
SCA12_PPP2R2B																		
SCA17_TBP																		
SCA36_NOP56									1.73E-02									

Count	Colour	Description
7		True positive (Significant in STRetch and pathogenic expansion in PCR)
64		True negative (Not significant in STRetch, normal length on PCR)
2		False negative (Not sigificant in STRetch, pathogenic expansion by PCR)
7		Likely false positive (Signfiicant in STRetch, no PCR confirmation)
3		Possible true positives (Signfiicant in STRetch, non-pathogenic expansion confirmed by PCR and/or ExpansionHunter)
315		Likely true negative (Not sigificant in STRetch, no PCR confirmation)

Table S3: Pathogenic STR loci, positions in hg19. Also available as the bed file hg19.STR_disease_loci.bed on Figshare along with the other reference data at https://figshare.com/s/1a39be9282c90c4860cd. FRDA is not annotated as an STR in the reference genome and so was excluded from most analyses.

Chromosome	Start	End	Disease	Gene
chr3	63898361	63898392	SCA7	ATXN7
chr3	128891419	128891502	DM2	ZNF9
chr4	3076604	3076695	HD	HTT
chr4	41747993	41748039	CCHS	PHOX2B
chr5	146258291	146258322	SCA12	PPP2R2B
chr6	16327865	16327955	SCA1	ATXN1
chr6	170870995	170871105	SCA17	TBP
chr9	27573482	27573544	FTDALS1	C9orf72
chr9	71652203	71652205	FRDA	FXN
chr12	7045880	7045938	DRPLA	ATN1
chr12	50898785	50898805	FRA12A	DIP2B
chr12	112036754	112036823	SCA2	ATXN2
chr13	70713484	70713561	SCA8	ATXN8/ATXN8OS
chr14	23790681	23790701	OPMD	PAPBN1
chr14	92537355	92537397	SCA3/MJD	ATXN3
chr16	87637889	87637935	HDL2	JPH3
chr19	13318673	13318712	SCA6	CACNA1A
chr19	46273462	46273524	DM1	DMPK
chr20	2633379	2633421	SCA36	NOP56
chr22	46191235	46191304	SCA10	ATXN10
chrX	66765159	66765261	SBMA	AR
chrX	146993555	146993629	FXTAS	FMR1
chrX	147582125	147582273	FRAXE	AFF2

Table S4: PacBio validation of STRetch calls on CHM1 and CHM13 mixed Illumina data. This table lists all the loci that were called significant by STRetch compared to the 97 reference controls.

gene	repeat unit	type	position	allele ref	allele PacBio	sample	allele STRetch	rank (reference control)	p-val (reference control)
HIST2H2BA	A	upstream	chr1:120898564-120898595	31	NA	CHM1-CHM13-mix2	50.7	11	3.84E-02
BC038779	AG	upstream	chr2:62892328-62892385	28.5	72.5	CHM1-CHM13-mix2	46.0	3	7.78E-07
LPP	AGATAT	intronic	chr3:188331032-188331103	11.8	13.8	CHM1-CHM13-mix1	15.8	7	4.83E-02
OPA1	AAAAG	intronic	chr3:193314083-193314212	25.8	31.2	CHM1-CHM13-mix2	30.8	7	4.05E-04
-	AAAAG	intergenic	chr4:99901543-99901596	10.8	24.8	CHM1-CHM13-mix1	15.6	5	1.23E-03
-	AAAAG	intergenic	chr4:99901543-99901596	10.8	24.8	CHM1-CHM13-mix2	15.8	8	4.05E-04
-	A	intergenic	chr5:60901988-60902016	28	NA	CHM1-CHM13-mix1	52.0	4	1.23E-03
MRDS1	AAG	intronic	chr6:9795375-9795631	86	98	CHM1-CHM13-mix1	94.0	3	8.01E-04
MRDS1	AAG	intronic	chr6:9795375-9795631	86	98	CHM1-CHM13-mix2	94.3	5	2.45E-04
GNA12	ACATC	intronic	chr7:2852270-2852331	12.2	28.2	CHM1-CHM13-mix2	31.9	9	1.12E-03
-	A	intergenic	chr9:76933570-76933599	29	262	CHM1-CHM13-mix1	78.1	2	2.56E-06
-	A	intergenic	chr9:76933570-76933599	29	262	CHM1-CHM13-mix2	69.3	4	5.73E-05
ABCA1	ACCCCC	intronic	chr9:107624982-107625036	9	18	CHM1-CHM13-mix2	14.0	6	4.05E-04
GABRG3	AGAT	intronic	chr15:27390742-27390811	17.8	23.55	CHM1-CHM13-mix1	23.8	6	3.50E-03
CHD3	CCG	intronic	chr17:7788625-7788663	12.7	30.7	CHM1-CHM13-mix1	24.9	1	1.88E-06
CHD3	CCG	intronic	chr17:7788625-7788663	12.7	30.7	CHM1-CHM13-mix2	27.9	2	3.62E-09
RNA5-8S5/ LOC100507412	ACCCC	intronic	chrUn_gl000220:120825-120864	7.8	NA	CHM1-CHM13-mix2	23.2	1	1.72E-16
RNA5-8S5/ LOC100507412	AGGC	intronic	chrUn_gl000220:121453-121502	12.2	NA	CHM1-CHM13-mix2	17.1	10	1.48E-02

Table S5: Primers used for amplification and sequencing of the AAACT repeat in an intron of the MTHFD2 gene (chr2:74430970-74431055).

	Primer name	Sequence (5'-3')	Position (chr2, hg19)	Length	%GC	Tm (°C)
**PCR**	MTHFD2 STR JC1 - FWD	TGGTGGGTGCCTGTATTCTCAG	+/ 74430660	22	54.5	58.1
	MTHFD2 STR JC2 - REV	TGCTTGAGGTCAGGAGTTCCAG	–/ 74431258	22	54.5	58
**Sequencing**	MTHFD2 STR JC1 seq - FWD	AAGAGGAGATTACTTCATTGGTC	+/ 74430873	23	39.1	51.8
	MTHFD2 STR JC2 seq - REV	CATGGCAAAACCCCGTCTCTG	–/ 74431223	21	57.1	58.1

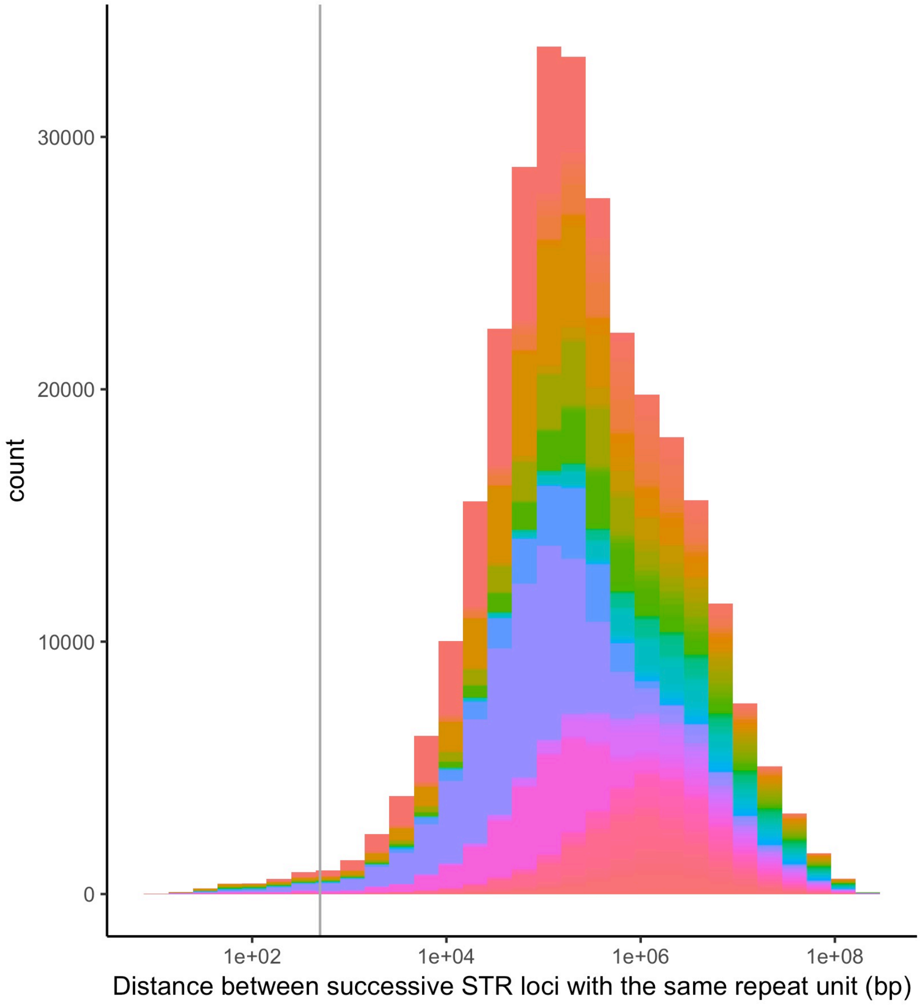

Fig. S1: The distribution of distances between STR loci with the same repeat unit in hg19. 0.93% of STR loci are within 500 bp of another STR locus with the same repeat unit (loci to the left of the solid vertical line at 500 bp). Colours indicate the 501 different STR repeat units.

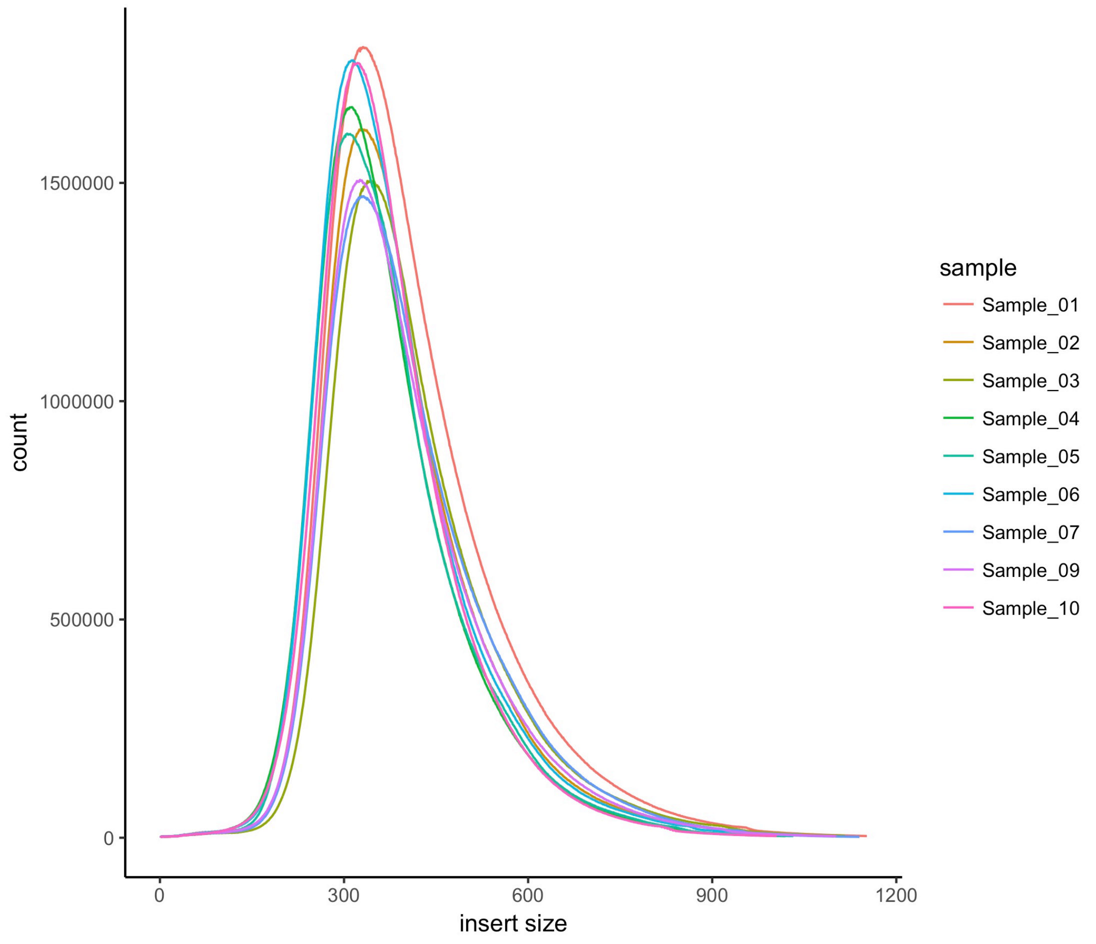

Fig. S2: Insert sizes of the ten true positive samples. The mean insert size ranged from 372 to 415 bp (standard deviation range: 130-150 bp).

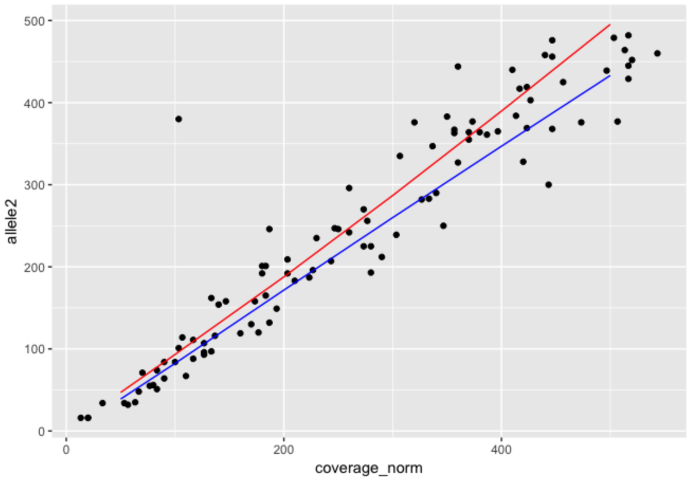

Fig. S3: The plot shows the simulated data (black points) with the upper (red) and lower (blue) bounds for the linear fit indicated. A plot of the number of reads mapping to the AGC decoy chromosome against the number of AGC repeat units inserted into the ATXN8 locus shows a clear linear relationship between these two variables.

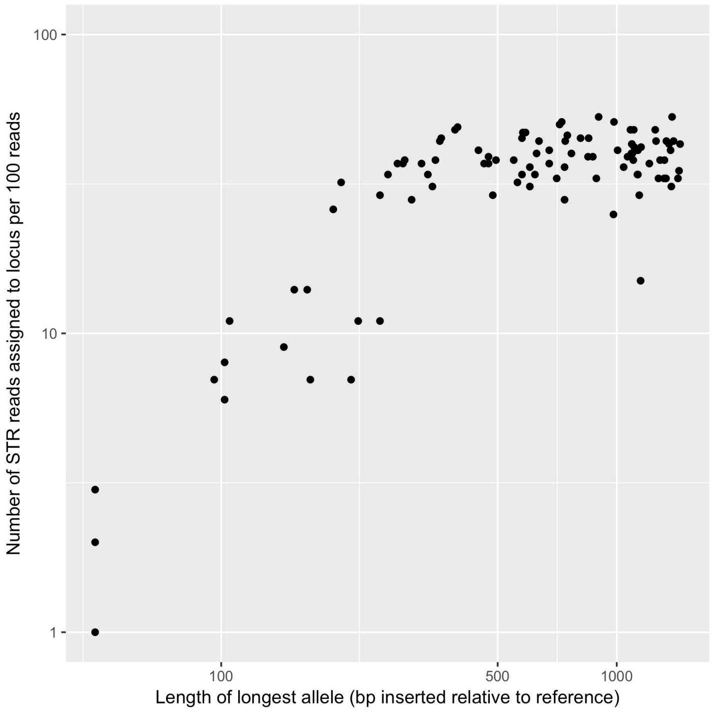

Fig. S4: The number of STR reads assigned to a locus does not increase beyond the insert size in simulated data, so STRetch will tend to underestimate alleles greater than the insert size.

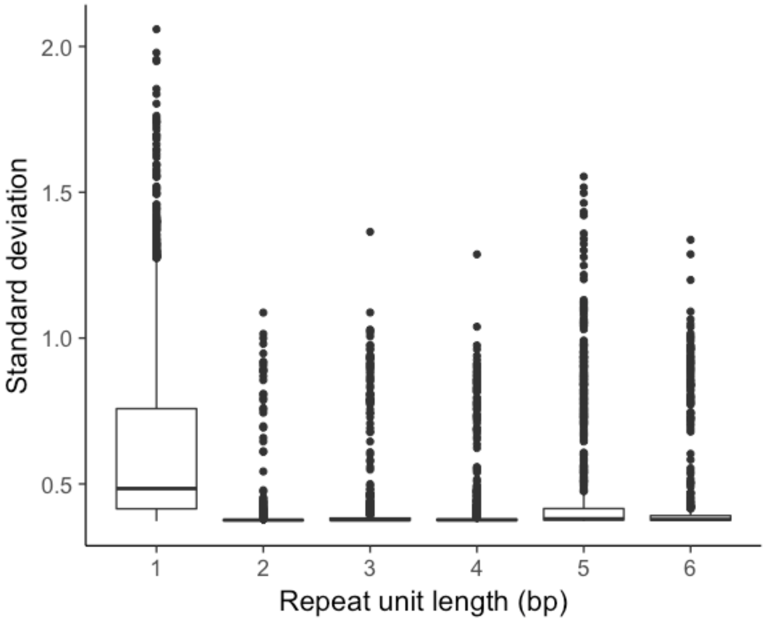

Fig. S5: Robust standard deviation of STR reads assigned to each locus across 97 WGS samples. Homopolymer (1 bp repeat unit) loci are the most variable between individuals.

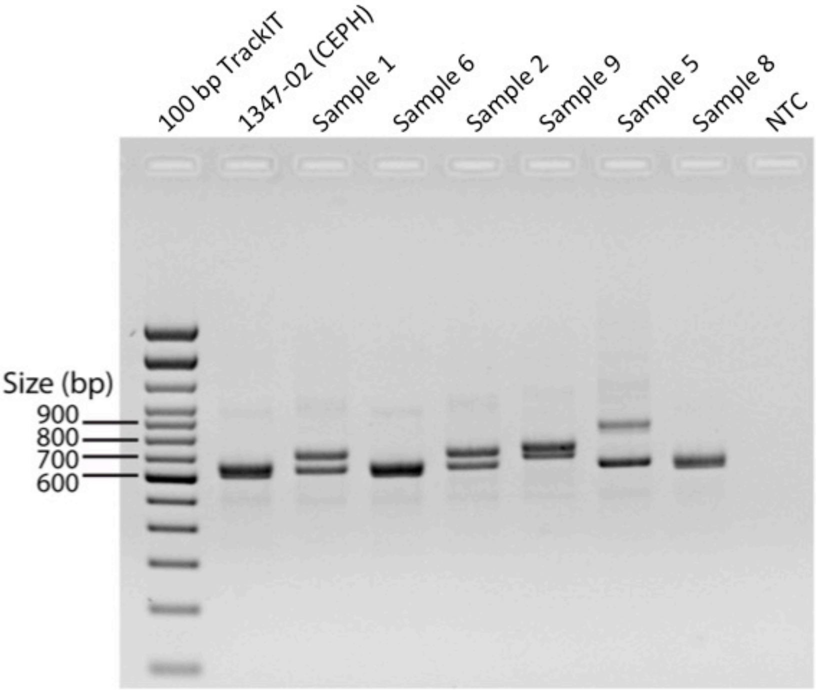

Fig. S6: PCR amplification of the AAACT repeat in an intron of the *MTHFD2* gene (chr2:74430970-74431055). Sample 5 is predicted by STRetch to have a significant expansion while Samples 1, 2, 6 and 8 are not. Sample 1347-02 (CEPH control DNA) has an unknown genotype at this locus. 2% agarose gel, see primer sequences in Supplementary Methods.

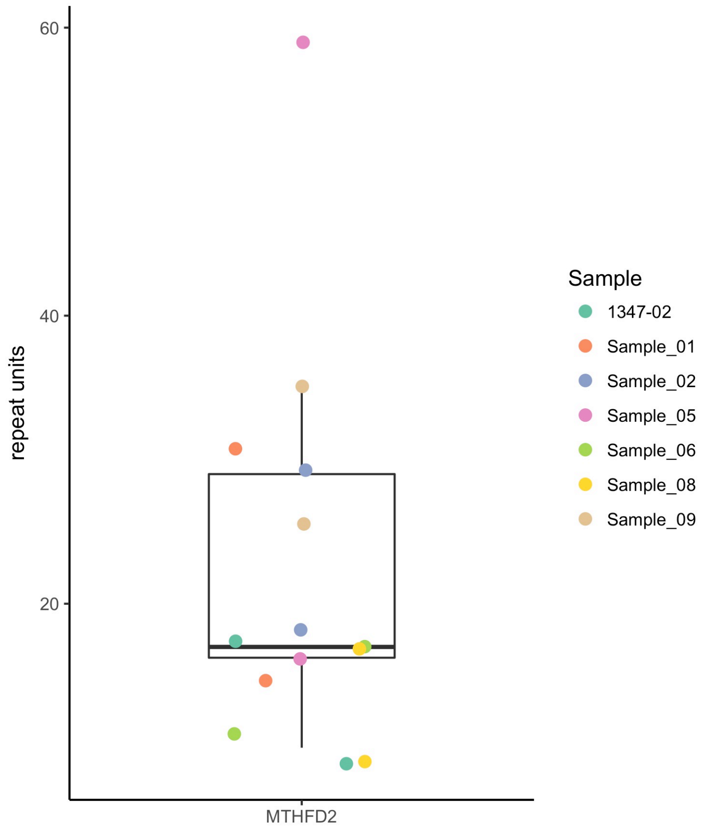

Fig. S7: Repeat sizes determined from Sanger sequencing of the AAACT repeat in an intron of the *MTHFD2* gene (chr2:74430970-74431055). Sample 5 is predicted by STRetch to have a significant expansion while Samples 1, 2, 6 and 8 are not. The 1347-02 control DNA sample has an unknown genotype at this locus. See primer sequences in Supplementary Methods.

Supplementary Methods

Runtime

In single-threaded mode STRetch takes approximately 3 hours, 50 minutes (of which 3 hours, 40 minutes is re-alignment) to perform analysis of a 40X PCR-free whole genome using an aligned cram file as input. This can be reduced to approximately 1 hour, 50 minutes by running STRetch with 12X concurrency. Samples can be run in parallel. STRetch is built on Bpipe, which interfaces with most common compute cluster environments.

ExpansionHunter custom repeat specifications (hg38):

```
{
    "RepeatId": "ATXN8",
    "RepeatUnit": "CTG",
    "CommonUnit": "true",
    "TargetRegion": "chr13:70139384-70139428"
}
{
    "RepeatId": "MTHFD2",
    "RepeatUnit": "TAGTT",
    "CommonUnit": "true",
    "TargetRegion": "chr2:74203844-74203928"
}
{
    "RepeatId": "NOP56",
    "RepeatUnit": "GGGCCT",
    "CommonUnit": "true",
    "TargetRegion": "chr20:2652733-2652775"
}
{
    "CommonUnit": "true",
    "RepeatId": "ZNF9",
    "RepeatUnit": "CAGG",
    "TargetRegion": "chr3:129172577-129172656"
}
```
